# Quantitative targeted proteomics for occult cancer screening in patients with unprovoked venous thromboembolism: results from the prospective PLATO-VTE study

**DOI:** 10.1016/j.rpth.2025.103018

**Published:** 2025-08-19

**Authors:** Noori A.M. Guman, Noémie Kraaijpoel, Frits I. Mulder, Marc Carrier, Luis Jara-Palomares, Marcello Di Nisio, Walter Ageno, Jan Beyer-Westendorf, Frederikus A. Klok, Thomas Vanassche, Johannes M.M.B. Otten, Benilde Cosmi, Mike J.L. Peters, Marije ten Wolde, Aurélien Delluc, Pieter W. Kamphuisen, Verónica Sánchez-López, Ettore Porreca, Jip Ramaker, Patrick M.M. Bossuyt, Harry R. Büller, Henri H. Versteeg, Bart J.M. van Vlijmen, Nick van Es, Yassene Mohammed

**Affiliations:** 1 Department of Vascular Medicine, Amsterdam Cardiovascular Sciences, Amsterdam University Medical Center, University of Amsterdam, Amsterdam, the Netherlands; 2 Amsterdam Cardiovascular Sciences, Pulmonary Hypertension and Thrombosis, Amsterdam, the Netherlands; 3 Department of Internal Medicine, Tergooi Medical Center, Hilversum, the Netherlands; 4 Department of Medicine, The Ottawa Hospital Research Institute, University of Ottawa, Ottawa, Ontario, Canada; 5 Medical-Surgical Unit of Respiratory Diseases, Hospital Universitario Virgen del Rocio, Seville, Spain; 6 Centro de Investigación Biomédica en Red de Enfermedades Respiratorias, Instituto de Salud Carlos III, Madrid, Spain; 7 Department of Medicine and Ageing Sciences, Gabriele D’Annunzio University, Chieti, Italy; 8 Department of Medicine and Surgery, University of Insubria, Varese, Italy; 9 Division Thrombosis and Hemostasis, Department of Medicine I, University Hospital “Carl Gustav Carus,” Dresden, Germany; 10 Department of Medicine—Thrombosis and Hemostasis, Leiden University Medical Center, Leiden, the Netherlands; 11 Department of Cardiovascular Sciences, University Hospitals Leuven, Leuven, Belgium; 12 Department of Internal Medicine, Meander Medisch Centrum, Amersfoort, the Netherlands; 13 Angiology and Blood Coagulation Unit, University of Bologna, Istituto di Ricovero e Cura a Carattere Scientifico Azienda Ospedaliero-Universitaria di Bologna, Bologna, Italy; 14 Department of Internal Medicine, University Medical Center Utrecht, Utrecht, the Netherlands; 15 Department of Internal Medicine, Flevo Hospital, Almere, the Netherlands; 16 Instituto de Biomedicina de Sevilla, Hospital Universitario Virgen del Rocio, Consejo Superior de Investigaciones Científicas, Universidad de Sevilla, Seville, Spain; 17 Departamento de Ciencias Sociales y de la Salud, Centro san Isidoro, Seville, Spain; 18 Department of Innovative Technologies in Medicine and Dentistry, University “G. d’Annunzio” of Chieti-Pescara, Chieti, Italy; 19 Department of Neurosurgery, Brain Tumor Center Amsterdam, Cancer Center Amsterdam, Amsterdam University Medical Center/Vrije Universiteit, Amsterdam, the Netherlands; 20 Department of Epidemiology and Data Science, Amsterdam Public Health, Amsterdam University Medical Center, University of Amsterdam, Amsterdam, the Netherlands; 21 Einthoven Laboratory for Vascular and Regenerative Medicine, Division of Thrombosis and Hemostasis, Department of Internal Medicine, Leiden University Medical Centre, Leiden, the Netherlands; 22 Center for Proteomics and Metabolomics, Leiden University Medical Center, Leiden, the Netherlands; 23 Proteomics Centre, University of Victoria, Victoria, British Columbia, Canada; 24 Gerald Bronfman Department of Oncology, McGill University, Montreal, Québec, Canada

**Keywords:** early detection of cancer, liquid biopsy, neoplasms, proteomics, venous thromboembolism

## Abstract

**Background:**

About 5% of patients with unprovoked venous thromboembolism (VTE) have occult cancer. Despite standard cancer screening, 50% of cancers remain undetected.

**Objectives:**

We used quantitative targeted proteomics to identify novel cancer biomarkers among patients with unprovoked VTE.

**Methods:**

Patients aged ≥40 years with a first unprovoked VTE and without a malignancy in the preceding 5 years were invited to an international prospective cohort study. Plasma samples were collected within 10 days after VTE. The primary outcome was an adjudicated cancer diagnosis during 12-month follow-up. Concentrations of 269 plasma proteins covering coagulation, complement, and cancer-associated pathways were measured using quantitative mass spectrometry-based targeted proteomics. In a nested case-control study, protein profiles of patients with cancer were compared with those of randomly sampled unique control patients (ratio 3:1). Proteins with an unadjusted *P* value < .05 and fold change ≥15% were combined in a multivariable logistic regression model. To address the variability in the obtained model, the protein selection and model-building approach were replicated in 250 bootstrap samples, and an optimism-adjusted c-statistic was calculated.

**Results:**

Of the 476 included participants, 28 (5.9%) were newly diagnosed with cancer. Plasma samples were available for 24 cases, which were compared with those of 75 control patients. Concentrations of P-selectin, β-2 microglobulin, complement component 7, intracellular adhesion molecule 1, and lumican were higher in cases than in controls, whereas coagulation factor (F)VII, FX, and FXII, β-Ala-His dipeptidase, and kalistatin were lower. The optimism-adjusted c-statistic of the multivariable logistic regression model including these proteins was 0.78 (95% CI, 0.70-0.87).

**Conclusion:**

Ten differentially abundant proteins were identified in patients with occult cancer, suggesting potential of plasma proteomic tests as novel biomarker for occult cancer in patients with unprovoked VTE.

## Essentials

• Previous data suggest that proteomics can detect plasma proteins associated with cancer.
• In PLATO-VTE, we applied targeted proteomics to plasma from patients with unprovoked VTE.
• Ten protein concentrations differed between patients with and without cancer at 1 year.
• Targeted proteomics may potentially improve screening for occult cancer after unprovoked VTE.

## 1 Background

Unprovoked venous thromboembolism (VTE) can be the first manifestation of occult cancer. The cumulative incidence of a cancer diagnosis is about 5% within 12 months after VTE diagnosis [1]. Timely detection, in particular of early-stage cancers, may improve morbidity and mortality outcomes. Despite routine cancer screening, which is usually limited to medical history taking, physical examination, routine blood tests, chest X-ray, and age- and sex-specific screening, about 50% of the occult cancers in patients with unprovoked VTE remain initially undetected [1]. More extensive cancer screening, including thoracoabdominal computed tomography, doubles the proportion of patients diagnosed with cancer in the period after VTE diagnosis, but this approach is not recommended given the lack of clear benefit for cancer-related survival, concerns about radiation exposure, and the burden of false-positive findings [[1], [2], [3]].

Emerging data have shown the potential of plasma cancer biomarkers, also known as liquid biopsies, which could provide a noninvasive and highly sensitive occult cancer screening tool for patients with unprovoked VTE [4]. Many proteins have been reported to discriminate between individuals with and without specific types of cancer [5]. Some of these are currently used in clinical practice for diagnostic and prognostic purposes, such as prostate-specific antigen and carcinoembryonal antigen. Unfortunately, these biomarkers only detect a limited number of cancer types and have poor accuracy for early cancer detection as stand-alone tests [6].

Novel mass spectrometry (MS)-based technologies allow for absolute quantitation of hundreds of proteins using as little as 5 to 10 μL of plasma [[7], [8], [9], [10]]. The objective of this predefined analysis of the PLATO-VTE cohort study [11] was to explore the feasibility of employing absolute quantitative targeted proteomics in patients with unprovoked VTE and identify potential plasma proteins as novel biomarkers for occult cancer.

## 2 Methods

### 2.1 Study design and patients

PLATO-VTE was a multinational prospective cohort study evaluating novel biomarkers for occult cancer in patients with unprovoked VTE. A detailed description of the study protocol [11] and first results [12] have been previously published. Eligible patients were aged ≥40 years and had a first unprovoked lower extremity deep vein thrombosis or pulmonary embolism. VTE was considered unprovoked in the absence of pregnancy or puerperium, immobilization for ≥3 days, surgery, hospitalization in the preceding 3 months, known genetic or acquired thrombophilia, or use of systemic estrogen therapy. Patients with a malignancy in the preceding 5 years were excluded.

Blood was collected in EDTA tubes within 10 days after VTE diagnosis, centrifuged at room temperature in 2 steps of 20 minutes (120 × *g* and 360 × *g*, respectively) to obtain platelet-poor plasma, and stored at −80 °C. The main outcome was a centrally adjudicated solid or hematological cancer diagnosis during 12 months of follow-up. In a nested case-control study, patients with cancer were compared with randomly selected unique controls from the cohort in a 3:1 ratio, as previously described [12]. Within 1 to 4 weeks after VTE diagnosis, patients underwent standard-of-care limited screening for cancer, which consisted of medical history taking, physical examination, blood work (complete blood count, liver and kidney function tests, and lactate dehydrogenase levels), chest X-ray, and additional age- and gender-specific tests (eg, mammography, prostate specific antigen, and fecal occult blood test) as per local protocol.

### 2.2 MS-based targeted proteomics

We used multiple reaction monitoring (MRM) assays, which were developed and validated at the University of Victoria Proteomics Centre, Victoria, British Columbia, Canada, and included internal standard peptides for 269 proteins [7,9,10,13,14]. The MRM assays are characterized according to Tier 2 Clinical Proteomic Tumor Analysis Consortium guidelines [15] and have been applied previously to analyze plasma samples [7,9,10,16]. A list of the peptides and proteins is provided in Supplementary Table 1. Concentrations of endogenous proteotypic peptides were measured by comparing their response in the MS with the response of heavy-labeled internal standard peptides spiked in the sample at known amount. The sample preparation protocol was developed previously [13,14,[17], [18], [19]] and is included in the Supplementary Methods.

### 2.3 Statistical analysis

Absolute protein concentrations were used for unsupervised cluster analysis and heatmap visualization after centering and scaling. Only proteins with plasma concentrations 50% above the lower limit of quantification in 90% of all samples measured were included. Differences between groups were tested using the unpaired Student’s *t*-test statistic. Fold changes (FCs) were calculated on a base 2 logarithmic scale after dividing the individual protein concentrations by the corresponding reference abundance of the protein. Proteins with an unadjusted *P* value < .05 and an absolute log_2_-FC of 15% in protein abundance were considered for further investigation and model building. The FC threshold of 15% was based on the variation in an overall MRM experiment [20]. This threshold reflects that about 70% of quantified proteins have a coefficient of variation of <25%. The baseline for our longitudinal comparisons was the corresponding healthy control protein abundance. The possible interplay between the differentially abundant proteins was evaluated using search tool for the retrieval of interacting genes/proteins (STRING) interaction network. Functional analyses were performed by Cytoscape [21] and the Cytoscape plugin GeneMANIA [22] to evaluate potentially perturbed pathways. Potentially discriminating proteins based on an unadjusted *P* value < .05 were assessed in multivariable logistic regression analysis. Performance of the generated model was evaluated by calculating c-statistics with 95% CIs using cross-validation with 100 iterations. Multiple models with varying ratios of training and testing sets were built, which included 80% to 20%, 70% to 30%, 60% to 40%, and 50% to 50% training-testing sets. In an additional analysis to address the variability of the obtained model, the protein selection and model-building approach were replicated in 250 bootstrap samples, based on guidance from the Transparent Reporting of a multivariable prediction model for Individual Prognosis or Diagnosis Statement [23]. In brief, we calculated the proportion of times each of the proteins identified as potentially discriminating in the original dataset was selected across all bootstrap samples. The c-statistic of the model built in each bootstrap sample was compared with the c-statistic of the original model by calculating the difference. The optimism-adjusted c-statistic of the original model was then obtained by subtracting the average of these differences across all bootstrap samples from the initially obtained c-statistic for the original model. All data analyses and visualization were performed using R (version 4.2.1, R Foundation for Statistical Computing, Vienna, Austria).

## 3 Results

A total of 476 patients were included between June 2016 and October 2020. At the 12-month follow-up, 28 (5.9%) were diagnosed with cancer. The type of cancer was a solid tumor in 22 (92%) patients. The most frequent cancer types were pancreatic (*n* = 4, 17%) and lung (*n* = 4, 17%). Of the patients with solid cancer, 14 (64%) had locoregional disease and 8 (36%) had metastatic disease. A total of 18 (75%) cancers were detected by means of limited cancer screening (ie, positive screening results either directly led to the cancer diagnosis or triggered more extensive screening tests).

Plasma samples were available for 24 cancer patients; 75 patients not diagnosed with cancer were randomly selected as controls (Table 1) [12]. Their median age was comparable (68 vs 66 years; *P* = .14), but cancer patients were more often female (58% vs 41%; *P* = .21). Patients diagnosed with cancer during follow-up more frequently had a history of cancer >5 years prior to inclusion (21% vs 3%; *P* = .01).

**Table 1:** Patient characteristics at unprovoked venous thromboembolism diagnosis.

Patient characteristics	Patients with cancer during 12-month follow-up *n*=24	Randomly selected controls without cancer *n*=75	*P* value
Age in (y), median (IQR)	68 (63-76)	66 (57-73)	.14
Female, *n* (%)	14 (58.3)	32 (41.0)	.21
Body mass index, median (IQR)	26.8 (25.8-29.8)	27.4 (24.8-32.4)	.86
Smoking, *n* (%)			.89
Current or former smoker	10 (41.7)	37 (47.5)	−
Never smoked	12 (50.0)	36 (46.2)	−
Unknown	2 (8.3)	5 (6.4)	−
Previous provoked VTE, *n* (%)^a^	4 (16.7)	3 (3.8)	.09
Index event, *n* (%)			.32
Deep vein thrombosis only	12 (50.0)	41 (52.6)	−
Pulmonary embolism only	6 (25.0)	27 (34.6)	−
Pulmonary embolism and deep vein thrombosis	6 (25.0)	10 (12.8)	−
Previous malignancy >5 y prior to enrolment, *n* (%)	5 (20.8)	2 (2.6)	.01
Limited cancer screening results raising cancer suspicion, *n* (%)	18 (75.0)	7 (9.0)	<.001
Time (d) between VTE diagnosis and blood withdrawal, median (IQR)	4.5 (1.0-7.2)	4.0 (1.0-7.0)	.52
Days from VTE to cancer diagnosis, median (IQR)	86 (40-157)	−	−
Solid cancer, *n* (%)	22 (91.7)	−	−
Locoregional disease	14 (63.6)	−	−
Distant metastases	8 (36.4)	−	−
Hematological cancer, *n* (%)	2 (8.3)	−	−
Cancer type, *n* (%)		−	−
Pancreatic	4 (16.0)	−	−
Lung (NSCLC)	3 (12.0)	−	−
Melanoma	2 (8.0)	−	−
Ovarian	2 (8.0)	−	−
Prostate	2 (8.0)	−	−
Renal	2 (8.0)	−	−
Breast	1 (4.0)	−	−
Colon	1 (4.0)	−	−
Esophageal	1 (4.0)	−	−
Leiomyosarcoma (pelvis)	1 (4.0)	−	−
Lung (carcinoid)	1 (4.0)	−	−
Dermatofibrosarcoma protuberans	1 (4.0)	−	−
Vaginal	1 (4.0)	−	−
Chronic lymphocytic leukemia	1 (4.0)	−	−
Follicular lymphoma	1 (4.0)	−	−

NSCLC, non small cell lung carcinoma; VTE, venous thromboembolism.

a Only 1 patient had a history of both cancer and VTE.

### 3.1 Discriminating proteins

**Figure 1:**
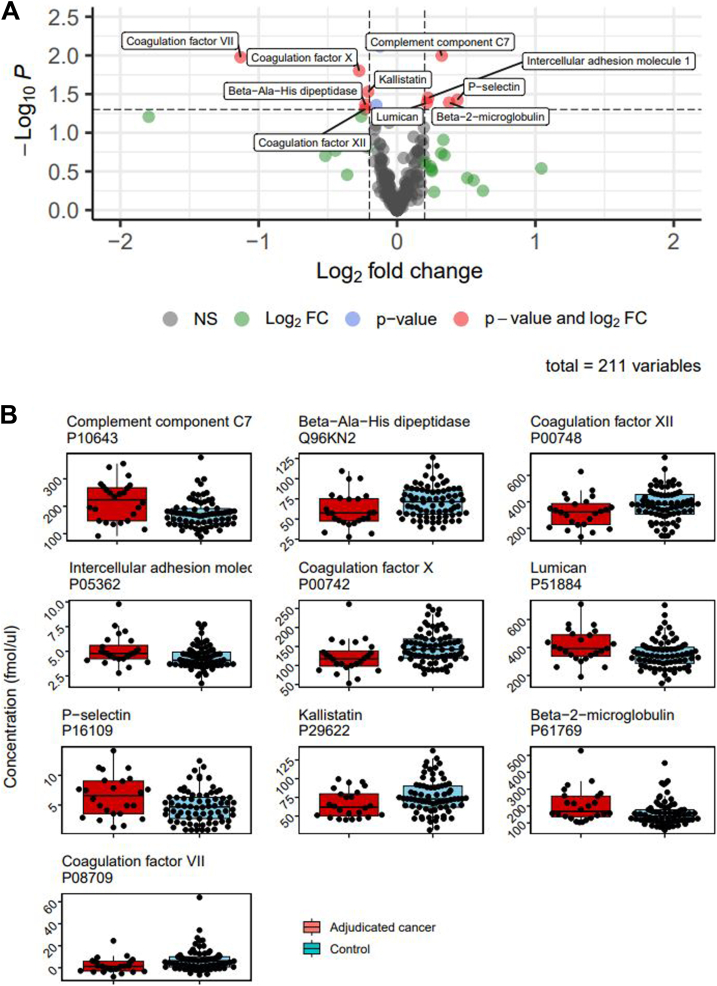
Proteins significantly differentiated between patients with unprovoked venous thromboembolism diagnosed with cancer during 12 months and selected controls without cancer. (A) Volcano plot. Each dot represents a measured protein with a log_2_-fold change (FC) in cases relative to controls on the x-axis and a –log_10_-fold *P* value for Student’s *t*-test on the y-axis. Proteins with *P* < .05 and an FC ≥15% were considered significantly differentiated and are presented in blue. (B) Plasma concentrations of the 9 differentially abundant proteins in cases and controls. NS, not significant.

**Figure 2:**
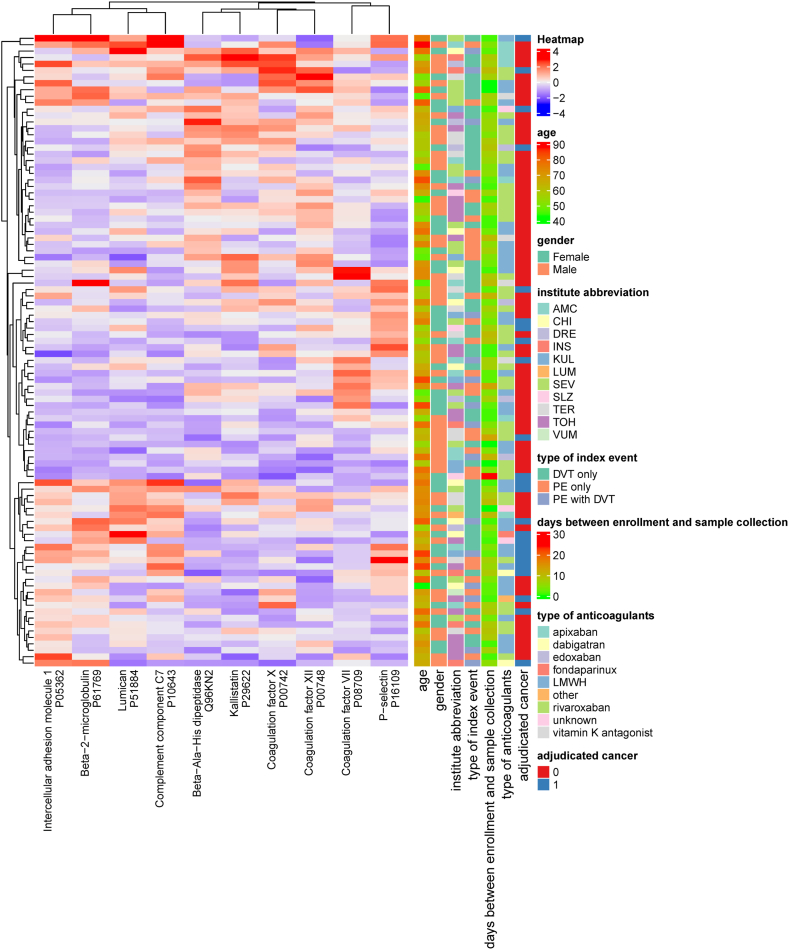
Heatmap of the 10 differentially abundant proteins. AMC, Academic Medical Center, Amsterdam, the Netherlands; CHI, Università degli Studi “Gabriele d'Annunzio”, Chieti, Italy; DVT, deep vein thrombosis; DRE, Universitätsklinikum Dresden, Dresden, Germany; INS, University of Insubria, Varese, Italy; KUL, Katholieke Universiteit Leuven, Leuven, Belgium; LUM, LUMC, Leiden, the Netherlands; LWMH, low-molecular-weight heparin; PE, pulmonary embolism; SEV, Hospital Universitario Virgen del Rocío: Sevilla, Andalucía, ES; SLZ, Slotervaartziekenhuis, Amsterdam, the Netherlands; TER, Tergooi hospital, Hilversum, the Netherlands; TOH, The Ottaway Hospital, Ottawa, Canada; VUM, VU medical center, Amsterdam, the Netherlands.

**Figure 3:**
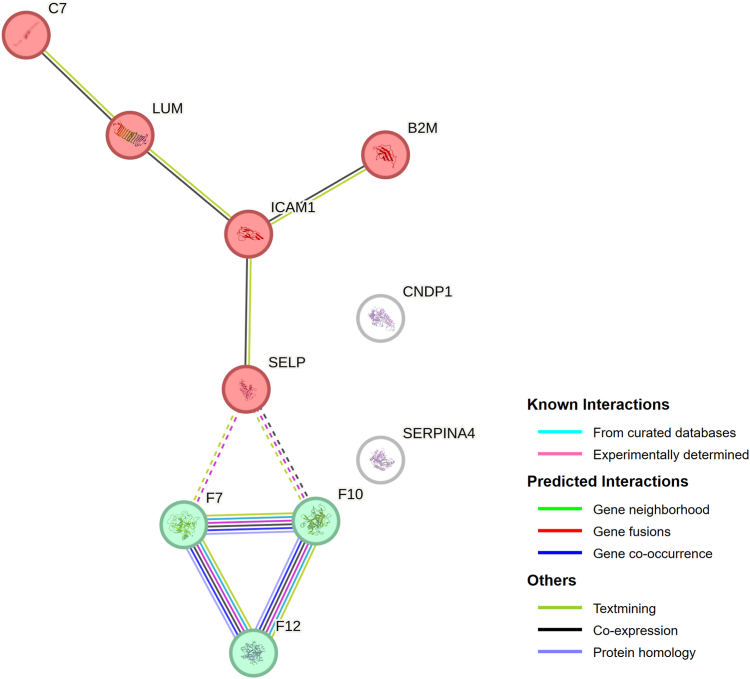
STRING interaction network of the 10 discriminating proteins. Two clusters arise: one is associated with blood coagulation and fibrin clot formation (in green), and the other has no known associations (in red). The type of relationship between the proteins is indicated in the legend. STRING, search tool for the retrieval of interacting genes/proteins.

**Figure 4:**
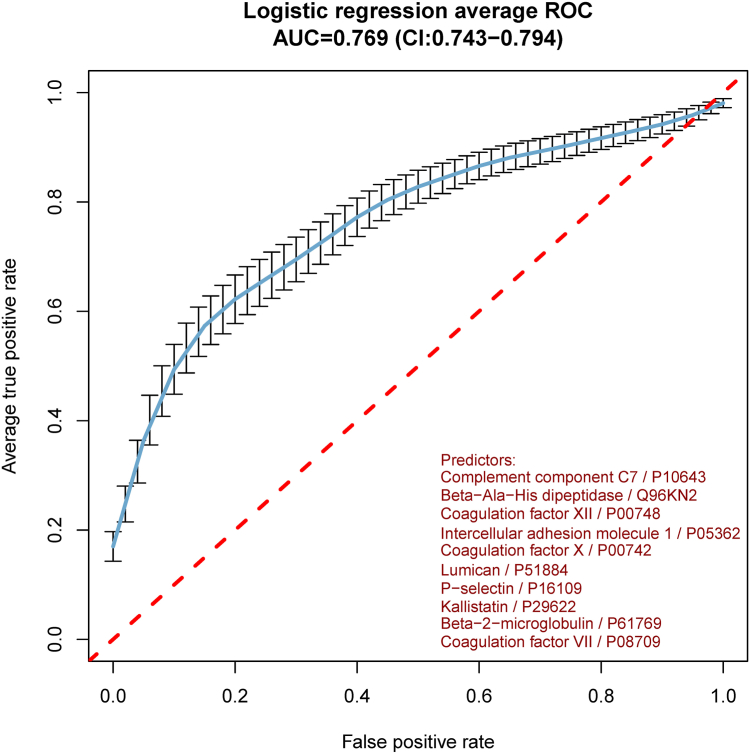
Receiver operating characteristic (ROC) curve of the multivariable regression analysis. AUC, area under the curve.

Of the 269 proteins measured with internal standards, 211 (77%) passed all quantifiability criteria and were considered for further comparison and analysis (Supplementary Table 1). The heatmap of the centered and scaled protein abundances in all samples is shown in Supplementary Figure 1. No clustering effects were observed for age, sex, participating hospitals, time between VTE diagnosis and sample collection, type of VTE, and type of anticoagulant therapy. Of the 211 proteins, 10 (4.7%) were considered differentially abundant based on a log_2_-FC >15% and an unadjusted *P* value < .05. P-selectin, β-2 microglobulin, complement component 7 (C7), intracellular adhesion molecule 1 (ICAM-1), and lumican concentrations were higher in cases compared with controls, while coagulation factor (F)VII, FX, and FXII, β-Ala-His dipeptidase, and kallistatin were lower in cases compared with controls (Figures 1 and 2). The STRING interaction network of these 10 proteins is shown in Figure 3, which shows the interplay of the proteins based on the current knowledge of the community. The STRING network analysis highlights 2 clusters: one is associated with blood coagulation (FVII, FX, and FXII), and the other (SELP, ICAM-1, B2M, LUM, and C7) represents associations that are mainly based on coexpression. When combining the 10 identified proteins in a multivariable logistic regression model, the cross-validated c-statistic was 0.78 (95% CI, 0.76-0.81; see Figure 4). Coefficients of the 10 proteins in the multivariable model are shown in Supplementary Table 2 and Supplementary Figure 2. When variable selection was repeated in the 250 bootstrap samples, the identified discriminating proteins recurred in 30% to 50% of the samples (Supplementary Table 2). The other quantified proteins recurred, on average, in 5.1% of regular bootstrapping. The optimism-adjusted c-statistic using the bootstrap method was 0.78 (95% CI, 0.70-0.87).

## 4 Discussion

Liquid biopsies can be used to detect circulating tumor-derived material with the advantages of being minimally invasive, affordable, and suitable for widespread use. In this exploratory analysis of the prospective PLATO-VTE cohort study, plasma concentrations of 211 quantifiable proteins were measured, focusing on those involved in coagulation, complement, and cancer-associated pathways. Ten proteins were considered differentially abundant in patients who were diagnosed with cancer during 12-month follow-up compared with those without a cancer diagnosis. Nine of 10 proteins are secreted proteins, while ICAM-1 is involved in cell adhesion and is usually found on the cell membrane. A multivariable logistic regression model incorporating these 10 proteins demonstrated good discrimination, with a c-statistic of 0.78 (95% CI, 0.76-0.81). These results suggest the potential utility of precise plasma protein concentrations as markers for screening occult cancer in patients with unprovoked VTE. Further research should focus on identifying predictive plasma proteins and validating these findings in larger cohorts.

The functions of the 10 differentially abundant proteins in physiological conditions are summarized in Table 2 [[24], [25], [26], [27], [28], [29], [30], [31], [32], [33], [34], [35], [36], [37]]. P-selectin, β-2 microglobulin, ICAM-1, and lumican have been extensively investigated in relation to various types of cancer [[24], [25], [26], [27], [28], [29],[33], [34], [35], [36],^38^]. Elevated P-selectin levels have shown associations with cancer progression and thromboembolic complications in cancer patients [[24], [25], [26]]. Similarly, elevated β-2 microglobulin levels have been linked to cancer progression [[27], [28], [29]]. The relationship between ICAM-1 and lumican and cancer appears to be variable, depending on the specific type and stage of cancer [[33], [34], [35], [36],^38^]. Limited data are available regarding the association of C7 and kallistatin with cancer; one prior study reported C7 gene polymorphisms in liver transplant recipients being linked to hepatocellular carcinoma recurrence after transplantation [30], while another study noted lower kallistatin levels in hepatocellular carcinoma patients compared with healthy controls [37]. The hypercoagulable state in patients with cancer compared with healthy controls is well established [39,40]. We speculated that lower values of coagulation FVII, FX, and FXII potentially indirectly reflect higher consumption of these proteins due to cancer-induced hypercoagulability. The potential influence of anticoagulant treatment was considered. However, only 3 patients were not receiving anticoagulation at the time of sampling, 2 of whom were later diagnosed with cancer. As the vast majority of both cases and controls were treated, and the median time between initiation of anticoagulation and sampling (median [IQR]) was comparable between the groups (5 [1–8] days vs 5 [2–7] days; *P* = .71), it is unlikely that anticoagulation accounts for the observed lower protein levels in patients with cancer. Lower β-Ala-His dipeptidase levels in patients with cancer compared with controls have not been described before. The presence of interaction clusters between selected discriminating proteins indicates potential coregulation or involvement in common signaling pathways, which may underpin the observed changes in protein abundance.

**Table 2:** Top discriminating proteins between patients with cancer and control patients.

Protein name	Log_2_-FC	Unadjusted *P* value	Function	Previous association with cancer
P-selectin	0.436	.04	A proteoglycan, which mediates rapid rolling of leukocytes over vascular surfaces during the initial steps in inflammation.	Elevated levels have been described in cancer patients and have been associated with cancer progression and thromboembolic complications [[24], [25], [26]].
β-2 microglobulin	0.377	.04	Component of the class I MHC. Involved in the presentation of peptide antigens to the immune system.	Elevated levels in patients with various types of cancer, and potentially associated with cancer prognosis [[27], [28], [29]].
C7	0.323	.01	Constituent of the membrane attack complex that plays a role in the innate and adaptive immune response.	C7 gene polymorphisms are associated with HCC recurrence after liver transplantation [30].
ICAM-1	0.225	.04	ICAM proteins are ligands for the leukocyte adhesion protein LFA-1.	Association with cancer varies depending on the type of cancer. In gastric and nasopharyngeal cancer, higher ICAM-1 expression was associated with more advanced stages and worse prognosis [31,32], whereas in breast cancer, it has been associated with better survival [33].
Lumican	0.215	.04	An extracellular matrix protein.	Association with cancer varies depending on the type of cancer. In advanced-stage colorectal cancer, lumican expression was found to be associated with worse prognosis [34], whereas in early-stage colorectal cancer and pancreatic ductal carcinoma, it has been associated with better prognosis [35,36].
Coagulation FVII	−1.762	.01	Initiates the extrinsic pathway of blood coagulation.	Not described before.
Coagulation FX	−0.274	.02	Vitamin K-dependent glycoprotein that converts prothrombin to thrombin.	Not described before.
Coagulation FXII	−0.230	.049	Participates in the initiation of blood coagulation, fibrinolysis, and the generation of bradykinin and angiotensin.	Not described before.
β-Ala-His dipeptidase	−0.229	.04	Catalyzes hydrolysis of carnosine.	Not described before.
Kallistatin	−0.208	.03	Inhibits human amidolytic and kininogenase activities of tissue kallikrein.	Lower in hepatocellular carcinoma patients than in healthy controls. A decrease in serum kallistatin levels appeared to reflect the extent of cirrhosis, with the lowest levels associated with higher grades of cirrhosis [37].

C7, complement component 7; FC, fold change; FVII/FX/FXII, factor VII/X/XII; HCC, hepatocellular carcinoma; ICAM, intracellular adhesion molecule; MHC major histocompability complex.

In the first analysis of the PLATO-VTE study [12], we found that 6% of patients with unprovoked VTE were diagnosed with cancer within 1 year, with 72% detected through limited screening. The lower-than-expected proportion of missed cancers may reflect heightened clinical awareness, more consistent implementation of screening strategies outlined in the study protocol, and the inclusion of patients with suspected but unconfirmed cancer at presentation [11]. However, approximately 30% of cancers still went undetected, which highlights the need for more sensitive diagnostic tools. The previous analysis of PLATO-VTE also evaluated platelet RNA sequencing for occult cancer screening, a technique that measured RNA expression profiles in platelets, which had been shown to discriminate between cancer patients and healthy individuals [12]. Unfortunately, the study demonstrated poor diagnostic accuracy of this test for occult cancer in patients with unprovoked VTE (area under the receiver operating curve of 0.54; 95% CI, 0.41-0.66). Compared with RNA sequencing, proteomics is more complex and cannot be applied on mass scale, but it is an accurate alternative for quantitation of proteins that are known to be involved in cancer-associated biological cellular pathways. When we compared the plasma proteomics abundance data with the platelet RNA sequencing results, we found poor correlation ranging from −0.66 to 0.50. This is not surprising given that these are 2 different compartments characterized by 2 different molecular contents. Although the quantitative proteomics approach used here primarily provided information on the protein abundance, the approach can be extended to provide additional in-depth information on the modification and function of the selected proteins [7], and thereby may potentially be a more sensitive screening tool.

Strengths of this current work include a predefined analysis plan in the original protocol of the PLATO-VTE study, a multicenter and international approach, uniform sample collection procedures across the different participating centers, blinding of laboratory personnel, and use of validated multiplexed assays to quantify proteins that largely cover the coagulation and complement cascade, as well as a few cancer-associated proteins. The main limitation is the limited number of cancer cases (*n* = 24) in relation to the number of proteins evaluated (*n* = 211), which may have resulted in type I errors when selecting the 10 differentially abundant proteins in univariable analyses. Such an effect was suggested by the sensitivity analysis, in which the selected proteins recurred in 30% to 50% of bootstrap samples. Nonetheless, the optimism-adjusted c-statistic of 0.78 suggests that plasma proteomics holds potential to discriminate between patients with vs those without cancer. External validation is needed to evaluate the performance of the 10 proteins in independent cohorts. The limited number of cancer cases hampered comparison of the value of proteomic biomarkers, in addition to limited cancer screening, as well as subgroup analyses of the different cancer types. The wide variety of cancer types in our study also hampered evaluation of cancer type-specific proteomic patterns. Blood samples were collected within 10 days after VTE diagnosis at baseline, some of which were collected after anticoagulant therapy was initiated. The extent to which timing within these 10 days, as well as anticoagulation, affected the measurements is unknown. No differences were observed in median time from VTE diagnosis to blood withdrawal in cases and controls. We did not collect data on ethnicity in this study, which may limit the extent to which we can explore the role of socio-cultural determinants of health. However, we expect that the influence of ethnic diversity on our findings is limited, since the study was conducted exclusively in European countries and Canada, where most participants were nonmigrants of predominantly European descent.

Proteomics-based technologies in cancer research have enabled the identification of cancer biomarkers and protein expression patterns that can be used to assess prognosis [41]. Proteomic tests are minimally invasive and feasible, which makes them an attractive alternative test for cancer screening. The findings of this study suggest that MS-based proteomics analysis can detect subtle differences in protein concentrations in patients with unprovoked VTE with and without occult cancer. Further, while we used targeted quantitative proteomics in our work here, it should also be possible to use other plasma quantification methods, like antibody-based methods, with which proteomics showed good correlations in previous studies. However, despite their promise, current MS-based approaches still face practical challenges that limit widespread clinical implementation. These include the need for specialized expertise, higher costs, and longer turnaround times compared with standard assays. Nonetheless, ongoing efforts to standardize workflows, automate sample preparation, and reduce costs [42,43], together with successful applications in Clinical Laboratory Improvement Amendments-certified laboratories [[44], [45], [46]], for example, assays for thyroglobulin and apolipoproteins, highlight the growing feasibility of this approach in clinical practice.

While the proteomic panel used in our study may not immediately indicate the site or type of malignancy, the presence of a cancer-related protein signature could serve as an early warning sign. This may prompt more intensive follow-up or targeted diagnostic evaluation to facilitate earlier cancer detection. Recent studies have demonstrated the capability of proteomic profiling to distinguish between tumor types and tissue origins [47,48]. Whether such specificity can be achieved in patients with unprovoked VTE remains an important question for future research. Ultimately, a deeper understanding of how these identified proteins interact with known clinical and laboratory risk factors for cancer is needed to fully assess the potential of proteomic analysis for occult cancer screening after unprovoked VTE.
