# Supplementary material for: Quantitative targeted proteomics for occult cancer screening in patients with unprovoked venous thromboembolism: results from the prospective PLATO-VTE study
Source: Res Pract Thromb Haemost. 2025 Aug 19;9(6):103018. doi: 10.1016/j.rpth.2025.103018 (PMC12478244; doi:10.1016/j.rpth.2025.103018)
Supplement: Supplementary Material [file mmc1.docx]

**Supplemental Methods**

***Sample preparation protocol***

Briefly, 10 μL of plasma was diluted with 20 μL of 9M urea/20mM dithiothreitol and incubated for 30 minutes at 37ºC to achieve denaturation and reduction. The samples were alkylated with iodoacetamide (40 mM final concentration) for 30 minutes at room temperature in the dark, and then the samples were diluted 10-fold in 100 mM Tris prior to tryptic digestion. Digestion was carried out at a 10:1 substrate:enzyme ratio using tosyl phenylalanyl chloromethyl ketone (TPCK)‐treated trypsin (Worthington) for 18 hours at 37ºC. After digestion, samples were acidified with aqueous 1% formic acid (FA), and a chilled stable isotope‐labeled standard (SIS) peptide mixture was added. Samples were concentrated via solid phase extraction (SPE; 10 mg Oasis HLB cartridges; Waters), using the manufacturer's recommended protocol. The SPE column was conditioned with 100% methanol (1 mL), followed by washing with 99.9% H_2_O/0.1% FA (1 mL), the sample (diluted to 1 mL using 99.9% H_2_O/0.1% FA) was then loaded onto the column, followed by washing two times with water (1 mL each). Finally, the sample was eluted with 55% acetonitrile /0.1% FA (300 μL) and lyophilized to dryness. The dried samples were rehydrated in 0.1% FA to a 1 μg/μL concentration for liquid chromatography (LC)/MRM‐MS analysis. The samples were separated on‐line with a reversed phase‐ultra high-performance liquid chromatography (RP‐UHPLC) column (EclipsePlusC18 RRHD 150 × 2.1 mm i.d., 1.8 μm particle diameter; Agilent) maintained at 50°C. Peptide separations were performed at 0.4 mL⁄min over a 56‐minute run, via a multi‐step LC gradient. The solvents were aqueous mobile phase – solvent A – with 0.1% formic acid in LC-MS grade water, and the organic mobile phase – solvent B – with 0.1% formic acid in LC-MS grade acetonitrile. The exact gradient was as follows (time point in minutes, solution B%): 0 min, 2%; 2 min, 7%; 50 min, 30%; 53 min, 45%, 53.5 min, 80%; 55.5 min, 80%; 56 min, 2%. A post‐column equilibration of 4 minutes was used after each sample analysis. The LC system was interfaced to a triple‐quadrupole mass spectrometer (Agilent 6490) via a standard‐flow electrospray ionization (ESI) source, operated in the positive ion mode. The MRM acquisition parameters employed for the quantitation were as follows: 3500 V capillary voltage, 300 V nozzle voltage, 11 L/min sheath gas flow at a temperature of 250°C, 15 L/min drying gas flow at a temperature of 150°C, 30 psi nebulizer gas pressure, 380 V fragmentor voltage, 5 V cell accelerator potential, and unit mass resolution in the first and third quadrupoles. For optimal peptide collision‐induced dissociation, peptide‐specific collision energy (CE) values had previously been determined experimentally. The exact CE value for each peptide is available from PeptideTracker^43^ (http://peptidetracker.proteincentre.com/).

We used the Skyline software to inspect the peptide response peaks and ensure accurate selection, retention time, integration, and uniformity of peak shape for the endogenous and internal standard peptide signals.^44^ For each peptide, the relative peak area ratio of the endogenous to the heavy labeled internal standard peptide was calculated. This ratio and the known concentration of internal standard peptide were used to calculate the concentration of the endogenous peptide in the sample by comparison to a standard curve generated in pooled sample. The criteria used for the standard curve regression analysis were 1/x2 regression weighting, <15% deviation in a given level's precision and accuracy for each concentration level, as well as 20% at the lower limit of quantification.

**Supplementary Table 1.** Targeted proteomics panel with protein detectability and quantifiability

| Protein | UniProtKB accession | Surrogate peptide | LLOQ (fmol/μL) | Mean of all measurements (fmol/μL) | Detected | Quantified (minimum 1% of measurements above LLOQ) | Measurements above 50% LLOQ |
| --- | --- | --- | --- | --- | --- | --- | --- |
| Transcription factor SOX-11 | P35716 | AAQSGDYGGAGDDYVLGSLR | 34.25 | -13.82 | No | No | 0 |
| Complement component C7 | P10643 | AASGTQNNVLR | 55.78 | 184.95 | Yes | Yes | 99 |
| Peroxiredoxin-1 | Q06830 | ADEGISFR | 30.57 | 6.5 | Yes | No | 0 |
| Histidine-rich glycoprotein | P04196 | ADLFYDVEALDLESPK | 29.65 | 1403.16 | Yes | Yes | 99 |
| L-selectin | P14151 | AEIEYLEK | 13.6 | 39.9 | Yes | Yes | 99 |
| Desmoplakin | P15924 | AELIVQPELK | 9.6 | 4.57 | Yes | No | 41 |
| Coagulation factor V | P12259 | AEVDDVIQVR | 70.23 | 40.99 | Yes | No | 76 |
| Apolipoprotein M | O95445 | AFLLTPR | 7.99 | 404.6 | Yes | Yes | 99 |
| Interstitial collagenase | P03956 | AFQLWSNVTPLTFTK | 13.61 | 2.21 | Yes | No | 0 |
| C-reactive protein | P02741 | AFVFPK | 6.27 | 662.36 | Yes | Yes | 99 |
| Gelsolin | P06396 | AGALNSNDAFVLK | 82.74 | 4975.5 | Yes | Yes | 99 |
| Carboxypeptidase N subunit 2 | P22792 | AGGSWDLAVQER | 131.05 | 300.4 | Yes | Yes | 98 |
| N-acetylmuramoyl-L-alanine amidase | Q96PD5 | AGLLRPDYALLGHR | 101.86 | 244.05 | Yes | Yes | 99 |
| Interleukin-10 | P22301 | AHVNSLGENLK | 17.23 | 4.27 | Yes | No | 1 |
| Cathelicidin antimicrobial peptide | P49913 | AIDGINQR | 37.35 | 27.47 | Yes | Yes | 98 |
| Alpha-2-macroglobulin | P01023 | AIGYLNTGYQR | 121.92 | 9154.85 | Yes | Yes | 99 |
| Cystatin-C | P01034 | ALDFAVGEYNK | 25.74 | 49.47 | Yes | Yes | 99 |
| Beta-Ala-His dipeptidase | Q96KN2 | ALEQDLPVNIK | 35.78 | 68.76 | Yes | Yes | 99 |
| Insulin-like growth factor-binding protein 1 | P08833 | ALPGEQQPLHALTR | 15.65 | 1.62 | Yes | No | 0 |
| Fructose-bisphosphate aldolase B | P05062 | ALQASALAAWGGK | 17.99 | 11.81 | Yes | Yes | 46 |
| Angiotensinogen | P01019 | ALQDQLVLVAAK | 61.02 | 763.39 | Yes | Yes | 99 |
| Neuropilin-2 | O60462 | ALQVVR | 7.88 | 67.86 | Yes | Yes | 99 |
| Autism susceptibility gene 2 protein | Q8WXX7 | ALSLASSSGSDK | 8.75 | -0.35 | No | No | 0 |
| Protein DJ-1 | Q99497 | ALVILAK | 2.01 | 1.85 | Yes | Yes | 82 |
| Apolipoprotein A-I | P02647 | ATEHLSTLSEK | 108.47 | 33739.04 | Yes | Yes | 99 |
| Β-2-glycoprotein 1 | P02749 | ATVVYQGER | 32.12 | 3641.23 | Yes | Yes | 99 |
| Di-N-acetylchitobiase | Q01459 | ATYIQNYR | 50.46 | 23.65 | Yes | No | 34 |
| Phospholipid transfer protein | P55058 | AVEPQLQEEER | 13.96 | 46.75 | Yes | Yes | 99 |
| Collagen alpha-1(XVIII) chain | P39060 | AVGLAGTFR | 12.32 | 5.29 | Yes | Yes | 28 |
| Matrix metalloproteinase-9 | P14780 | AVIDDAFAR | 8.41 | 1 | Yes | No | 2 |
| Thyroxine-binding globulin | P05543 | AVLHIGEK | 5.2 | 266.21 | Yes | Yes | 99 |
| Xaa-Pro dipeptidase | P12955 | AVYEAVLR | 5.22 | 8.27 | Yes | Yes | 99 |
| Lysozyme C | P61626 | AWVAWR | 4.31 | 77.4 | Yes | Yes | 99 |
| Pappalysin-1 | Q13219 | AYLDVNELK | 3.18 | 0.31 | Yes | No | 0 |
| Afamin | P43652 | DADPDTFFAK | 55.21 | 391.57 | Yes | Yes | 99 |
| Antithrombin-III | P01008 | DDLYVSDAFHK | 177.44 | 23834.18 | Yes | Yes | 99 |
| Tumor necrosis factor receptor superfamily member 1B | P20333 | DEQVPFSK | 18.84 | 3.01 | Yes | No | 0 |
| Vitamin K-dependent protein Z variant 1 | P22891 | DFAEHLLIPR | 30.09 | 18.04 | Yes | Yes | 66 |
| Serotransferrin | P02787 | DGAGDVAFVK | 232.82 | 16951.88 | Yes | Yes | 99 |
| Haptoglobin | P00738 | DIAPTLTLYVGK | 377.83 | 38648.02 | Yes | Yes | 99 |
| Angiogenin | P03950 | DINTFIHGNK | 4.22 | 0.95 | Yes | No | 0 |
| Kininogen-1 | P01042 | DIPTNSPELEETLTHTITK | 39.98 | 1857.03 | Yes | Yes | 99 |
| Creatine kinase B-type | P12277 | DLFDPIIEDR | 5.56 | -0.08 | No | No | 0 |
| Complement component C6 | P13671 | DLHLSDVFLK | 220.89 | 216.31 | Yes | Yes | 95 |
| Vascular endothelial growth factor D | O43915 | DLIQHPK | 23.64 | 0 | No | No | 0 |
| Leucine-rich alpha-2-glycoprotein | P02750 | DLLLPQPDLR | 56.39 | 626.14 | Yes | Yes | 99 |
| Protein S100-A9 | P06702 | DLQNFLK | 10.29 | 35.26 | Yes | Yes | 98 |
| Angiopoietin-related protein 3 | Q9Y5C1 | DLVFSTWDHK | 73.56 | 33.84 | Yes | No | 24 |
| Proteoglycan 4 | Q92954 | DQYYNIDVPSR | 17.74 | 58.26 | Yes | Yes | 99 |
| Probable G-protein coupled receptor | Q8IZF2 | DVIVHPLPLK | 1.43 | 2.47 | Yes | Yes | 97 |
| Microtubule-associated protein tau | P10636 | EADLPEPSEK | 35.64 | 6.62 | Yes | No | 0 |
| Serum amyloid A-1 protein | P0DJI8 & P0DJI9 | EANYIGSDK | 11.15 | 1988.79 | Yes | Yes | 99 |
| Plasminogen | P00747 | EAQLPVIENK | 52.69 | 1619.2 | Yes | Yes | 99 |
| Tumor necrosis factor receptor superfamily member 1A | P19438 | EATLELLGR | 15.75 | 7.71 | Yes | No | 41 |
| C4b-binding protein alpha chain | P04003 | EDVYVVGTVLR | 29.06 | 3233.22 | Yes | Yes | 99 |
| Complement factor B | P00751 (not in isoform 2) | EELLPAQDIK | 11.57 | 1868.38 | Yes | Yes | 99 |
| Platelet glycoprotein VI | Q9HCN6 | EGDPAPYK | 28.66 | 1.4 | Yes | No | 0 |
| cDNA FLJ53327 highly similar to Gelsolin | P06396 | EGGQTAPASTR | 66.44 | 775.31 | Yes | Yes | 99 |
| Alpha-1-antichymotrypsin | P01011 | EIGELYLPK | 26.98 | 4891.82 | Yes | Yes | 99 |
| Zinc-alpha-2-glycoprotein | P25311 | EIPAWVPFDPAAQITK | 34.58 | 495.6 | Yes | Yes | 99 |
| Clusterin | P10909 | ELDESLQVAER | 58.6 | 2502.59 | Yes | Yes | 99 |
| Cadherin-5 | P33151 | ELDSTGTPTGK | 55.11 | 38.91 | Yes | Yes | 87 |
| Mucin-16 | Q8WXI7 | ELGPYTLDR | 29.05 | 11.07 | Yes | No | 16 |
| Prothrombin | P00734 | ELLESYIDGR | 70.47 | 3817.8 | Yes | Yes | 99 |
| Proenkephalin-A | P01210 | ELLETGDNR | 3.25 | 1.58 | Yes | No | 34 |
| Apolipoprotein C-IV | P55056 | ELLETVVNR | 25.3 | 43.23 | Yes | Yes | 93 |
| Chromogranin-A | P10645 | ELQDLALQGAK | 28.52 | 4.18 | Yes | No | 2 |
| Sterile alpha motif domain-containing protein 9-like | Q8IVG5 | ENVLDEVANAK | 18.57 | 0.22 | Yes | No | 0 |
| Glutamate receptor ionotropic NMDA 2B | Q13224 | EPGGPSFTIGK | 19.75 | 11.76 | Yes | No | 77 |
| Protein S100-B | P04271 | EQEVVDK | 1257.2 | 217.23 | Yes | No | 6 |
| Coagulation factor XII | P00748 | EQPPSLTR | 7.99 | 364.66 | Yes | Yes | 99 |
| Ras GTPase-activating protein nGAP | Q9UJF2 | ETQSTPQSAPQVR | 34.98 | 2.94 | Yes | No | 0 |
| Protein Z-dependent protease inhibitor | Q9UK55 | ETSNFGFSLLR | 52.05 | 41.07 | Yes | Yes | 90 |
| Natriuretic peptides B | P16860 | EVATEGIR | 8.57 | 3.62 | Yes | No | 12 |
| Apolipoprotein C-I | P02654 | EWFSETFQK | 148.81 | 3867.62 | Yes | Yes | 99 |
| Metalloproteinase inhibitor 2 | P16035 | EYLIAGK | 4.97 | 3.8 | Yes | Yes | 79 |
| Vitronectin | P04004 | FEDGVLDPDYPR | 160.79 | 2819.92 | Yes | Yes | 99 |
| Creatine kinase M-type | P06732 | FEEILTR | 6.33 | 4.1 | Yes | Yes | 80 |
| Interleukin-6 | P05231 | FESSEEQAR | 71.76 | 20.46 | Yes | No | 0 |
| Phosphatidylinositol-glycan-specific phospholipase D | P80108 | FGSSLITVR | 11.5 | 61.92 | Yes | Yes | 99 |
| Calcitonin | P01258 | FHTFPQTAIGVGAPGK | 7.38 | 4.64 | Yes | No | 81 |
| Insulin-like growth factor-binding protein 3 | P17936 | FLNVLSPR | 6.52 | 29.45 | Yes | Yes | 99 |
| Apolipoprotein B-100 | P04114 | FPEVDVLTK | 7.4 | 319.38 | Yes | Yes | 99 |
| Plasma protease C1 inhibitor | P05155 | FQPTLLTLPR | 30.11 | 2263.77 | Yes | Yes | 99 |
| Complement C1q subcomponent subunit C | P02747 | FQSVFTVTR | 16.77 | 706.23 | Yes | Yes | 99 |
| Thyroglobulin | P01266 | FSPDDSAGASALLR | 25.16 | 8.76 | Yes | No | 0 |
| Alpha-2-HS-glycoprotein | P02765 | FSVVYAK | 32.05 | 3661.62 | Yes | Yes | 99 |
| Glutamate receptor ionotropic NMDA 2ª | Q12879 | FSYIPEAK | 60.58 | 13.05 | Yes | No | 0 |
| Tenascin | P24821 | FTTDLDSPR | 11.1 | 4.86 | Yes | Yes | 29 |
| Tissue factor pathway inhibitor (isoform 1) | P10646-1 | FYYNSVIGK | 16.55 | 1.2 | Yes | No | 0 |
| IgGFc-binding protein | Q9Y6R7 | GATTSPGVYELSSR | 10.03 | 15.46 | Yes | Yes | 97 |
| Osteopontin | P10451 | GDSVVYGLR | 26.87 | 11.65 | Yes | No | 22 |
| Immunoglobulin heavy constant mu | P01871 | GFPSVLR | 43.08 | 5167.12 | Yes | Yes | 99 |
| Metalloproteinase inhibitor 1 | P01033 | GFQALGDAADIR | 32.05 | 10.56 | Yes | No | 6 |
| Plasma serine protease inhibitor | P05154 | GFQQLLQELNQPR | 14.15 | 86.97 | Yes | Yes | 97 |
| Transferrin receptor protein 1 | P02786 | GFVEPDHYVVVGAQR | 27.99 | 13.05 | Yes | No | 31 |
| Insulin-like growth factor I | P05019 | GFYFNKPTGYGSSSR | 123.63 | 40.99 | Yes | No | 0 |
| Collagen alpha-1(III) chain | P02461 | GGAGPPGPEGGK | 73.12 | 14.84 | Yes | No | 0 |
| Protein S100-A12 | P80511 | GHFDTLSK | 6.04 | 0.95 | Yes | Yes | 14 |
| Cation-independent mannose-6-phosphate receptor | P11717 | GHQAFDVGQPR | 8.75 | 4.64 | Yes | No | 57 |
| 60 kDa heat shock protein, mitochondrial | P10809 | GIIDPTK | 4.4 | 1.13 | Yes | No | 0 |
| Peroxiredoxin-2 | P32119 | GLFIIDGK | 13.17 | 16.33 | Yes | Yes | 95 |
| Vitamin K-dependent protein Z | P22891 | GLLSGWAR | 44.91 | 38.13 | Yes | Yes | 84 |
| Complement C1r subcomponent | P00736 | GLTLHLK | 15.78 | 415.18 | Yes | Yes | 99 |
| Serum amyloid A-4 protein | P35542 | GNYDAAQR | 29.34 | 1819.43 | Yes | Yes | 99 |
| Ig gamma-1 chain C region | P01857 | GPSVFPLAPSSK | 52.46 | 21720.09 | Yes | Yes | 99 |
| Hornerin | Q86YZ3 | GSGSGQSPSSGQHGTGFGR | 73.73 | 14.32 | Yes | No | 0 |
| Dickkopf-related protein 1 | O94907 | GSHGLEIFQR | 22.92 | 4.6 | Yes | No | 0 |
| Inter-alpha-trypsin inhibitor heavy chain H1 | P19827 | GSLVQASEANLQAAQDFVR | 3450.5 | 4075.34 | Yes | Yes | 98 |
| Transthyretin | P02766 | GSPAINVAVHVFR | 33.54 | 315.61 | Yes | Yes | 99 |
| Platelet-activating factor acetylhydrolase | Q13093 | GSVHQNFADFTFATGK | 67.46 | 21.09 | Yes | No | 0 |
| Ficolin-2 | Q15485 | GTHGSFANGINWK | 55.46 | 44.05 | Yes | Yes | 86 |
| Thrombospondin-1 | P07996 | GTLLALER | 11.21 | 29.61 | Yes | Yes | 97 |
| Coagulation factor XIII A chain | P00488 | GTYIPVPIVSELQSGK | 17.12 | 63.52 | Yes | Yes | 99 |
| Apolipoprotein(a) | P08519 | GTYSTTVTGR | 72.03 | 419.84 | Yes | Yes | 88 |
| Cartilage acidic protein 1 | Q9NQ79 | GVASLFAGR | 12.51 | 18.35 | Yes | Yes | 95 |
| Myelin basic protein | P02686 (not in isoform 2) | GVDAQGTLSK | 14.88 | 0.12 | Yes | No | 0 |
| Cholesteryl ester transfer protein | P11597 | GVSLFDIINPEIITR | 55.1 | 14.72 | Yes | No | 0 |
| Collagen alpha-1(I) chain | P02452 | GVVGLPGQR | 14.66 | 4.17 | Yes | No | 0 |
| Collagen alpha-2(I) chain | P08123 | GVVGPQGAR | 3.53 | -0.68 | No | No | 0 |
| Apolipoprotein C-III | P02656 | GWVTDGFSSLK | 56.86 | 6549.06 | Yes | Yes | 99 |
| Complement C2 | P06681 | HAFILQDTK | 25.42 | 121.06 | Yes | Yes | 99 |
| Protein AMBP | P02760 | HHGPTITAK | 82.81 | 689.65 | Yes | Yes | 99 |
| Fibrinogen β chain | P02675 | HQLYIDETVNSNIPTNLR | 117.32 | 24258.41 | Yes | Yes | 99 |
| Fibronectin | P02751 | HTSVQTTSSGSGPFTDVR | 116.59 | 669.35 | Yes | Yes | 99 |
| Complement C1q subcomponent subunit B | P02746 | IAFSATR | 5.63 | 384 | Yes | Yes | 99 |
| Galectin-3 | P17931 | IALDFQR | 7.34 | 1.6 | Yes | No | 4 |
| Carboxypeptidase B2 | Q96IY4 | IAWHVIR | 39.84 | 88.02 | Yes | Yes | 99 |
| 72 KDa type IV collagenase | P08253 | IDAVYEAPQEEK | 18.5 | 5.66 | Yes | No | 1 |
| Prolactin | P01236 | IDNYLK | 93.59 | 3.79 | Yes | No | 0 |
| Serum paraoxonase/arylesterase 1 | P27169 | IFFYDSENPPASEVLR | 118.24 | 833.97 | Yes | Yes | 99 |
| Adiponectin | Q15848 | IFYNQQNHYDGSTGK | 16.77 | 104.01 | Yes | Yes | 99 |
| A disintegrin and metalloproteinase with thrombospondin motifs 2 | O95450 | IILLSYGK | 4.15 | 0.74 | Yes | No | 2 |
| von Willebrand factor | P04275 | ILAGPAGDSNVVK | 24.74 | 15.6 | Yes | Yes | 51 |
| Serum paraoxonase/lactonase 3 | Q15166 | ILIGTVFHK | 3.3 | 7.3 | Yes | Yes | 98 |
| Tenascin-X | P22105 | ILISGLEPSTPYR | 9.09 | 5.5 | Yes | Yes | 77 |
| Cadherin-13 | P55290 | INENTGSVSVTR | 57.65 | 15.33 | Yes | No | 1 |
| A disintegrin and metalloproteinase with thrombospondin motifs 20 | P59510 | IPAGATNVDIR | 9.9 | 0.78 | Yes | No | 0 |
| Epidermal growth factor receptor | P00533 | IPLENLQIIR | 10.75 | 3.41 | Yes | No | 0 |
| Resistin | Q9HD89 | IQEVAGSLIFR | 2.79 | 1.45 | Yes | No | 54 |
| Coagulation factor XIII B chain | P05160 | IQTHSTTYR | 37.89 | 101.05 | Yes | Yes | 99 |
| Pregnancy zone protein | P20742 | ISEITNIVSK | 20.15 | 82.06 | Yes | Yes | 70 |
| Plastin-2 | P13796 | ISFDEFIK | 40.06 | 14.24 | Yes | No | 5 |
| Atrial natriuretic peptide receptor 1 | P16066 | ITDYGLESFR | 16.1 | 2 | Yes | No | 0 |
| Lipopolysaccharide-binding protein | P18428 | ITLPDFTGDLR | 70.6 | 135.53 | Yes | Yes | 99 |
| Neutrophil gelatinase-associated lipocalin | P80188 | ITLYGR | 4.06 | 3.42 | Yes | Yes | 88 |
| 78 kDa glucose-regulated protein | P11021 | ITPSYVAFTPEGER | 59.28 | 21.41 | Yes | No | 0 |
| Serum amyloid P-component | P02743 | IVLGQEQDSYGGK | 58.69 | 910.7 | Yes | Yes | 99 |
| Ceruloplasmin | P00450 | IYHSHIDAPK | 114.1 | 2159.86 | Yes | Yes | 99 |
| Target of Nesh-SH3 | Q7Z7G0 | IYLSDSLTGK | 8.92 | 8.79 | Yes | Yes | 98 |
| Thrombospondin-4 | P35443 | KPQDFLEELK | 34.03 | 4.5 | Yes | No | 0 |
| Glial fibrillary acidic protein | P14136 | LADVYQAELR | 6.64 | 1.65 | Yes | No | 0 |
| Insulin-like growth factor-binding protein complex acid labile subunit | P35858 | LAELPADALGPLQR | 6.72 | 88.44 | Yes | Yes | 99 |
| ADM | P35318 | LDVASEFR | 15.09 | 5.41 | Yes | No | 1 |
| SPARC | P09486 | LEAGDHPVELLAR | 8.18 | 7.72 | Yes | Yes | 91 |
| Oxidized low-density lipoprotein receptor 1 | P78380 | LEGQISAR | 8.99 | 1.39 | Yes | No | 6 |
| Alpha-1B-glycoprotein | P04217 (not in isoform 2) | LETPDFQLFK | 63.53 | 2336.59 | Yes | Yes | 98 |
| Heat shock protein β-1 | P04792 | LFDQAFGLPR | 66.15 | 16.87 | Yes | No | 0 |
| Apolipoprotein A-IV | P06727 | LGEVNTYAGDLQK | 28.98 | 1286.12 | Yes | Yes | 99 |
| Vitamin K-dependent protein C | P04070 | LGEYDLR | 24 | 33.91 | Yes | Yes | 97 |
| Ferritin light chain | P02792 | LGGPEAGLGEYLFER | 12.06 | 2.21 | Yes | No | 3 |
| Tumor necrosis factor receptor superfamily member 1A | P19438 | LGLSDHEIDR | 8.62 | 2.13 | Yes | No | 1 |
| Alpha-2-antiplasmin | P08697 (not in isoform 2) | LGNQEPGGQTALK | 35.4 | 913.83 | Yes | Yes | 99 |
| Apolipoprotein E | P02649 | LGPLVEQGR | 13.46 | 817.32 | Yes | Yes | 99 |
| Coagulation factor VIII | P00451 | LHPTHYSIR | 87.14 | 8.95 | Yes | No | 0 |
| Insulin-like growth factor-binding protein 2 | P18065 | LIQGAPTIR | 10.27 | 12.5 | Yes | Yes | 95 |
| Adipocyte plasma membrane-associated protein | Q9HDC9 | LLEYDTVTR | 12.77 | 24.06 | Yes | Yes | 98 |
| Intercellular adhesion molecule 1 | P05362 | LLGIETPLPK | 9.16 | 4.55 | Yes | Yes | 42 |
| Elastin | P15502 | LPGGYGLPYTTGK | 42.45 | 11.06 | Yes | No | 0 |
| Pigment epithelium-derived factor | P36955 | LQSLFDSPDFSK | 54.59 | 321 | Yes | Yes | 99 |
| Complement component C9 | P02748 | LSPIYNLVPVK | 57.19 | 757.45 | Yes | Yes | 99 |
| B-cell scaffold protein with ankyrin repeats | Q8NDB2 | LTIVHHPGGK | 11.98 | -1.28 | No | No | 1 |
| CD5 antigen-like | O43866 | LVGGLHR | 41.78 | 408.78 | Yes | Yes | 99 |
| Serum albumin | P02768 (not in isoform 2) | LVNEVTEFAK | 1149 | 620608.9 | Yes | Yes | 99 |
| Myeloblastin | P24158 | LVNVVLGAHNVR | 65.28 | 14.96 | Yes | No | 0 |
| Endothelial lipase | Q9Y5X9 | LVSALHTR | 1.47 | -0.36 | No | No | 0 |
| Fetuin-B | Q9UGM5 | LVVLPFPK | 54.51 | 48.98 | Yes | Yes | 94 |
| A disintegrin and metalloproteinase with thrombospondin motifs 9 | Q9P2N4 | LYNPDVR | 16.68 | 3.5 | Yes | No | 0 |
| Complement component C8 alpha chain | P07357 | MESLGITSR | 31.76 | 281.96 | Yes | Yes | 99 |
| Coagulation factor X | P00742 | MLEVPYVDR | 37.04 | 141.67 | Yes | Yes | 99 |
| Matrix Gla protein | P08493 | NANTFISPQQR | 51.75 | 20.16 | Yes | Yes | 19 |
| Hemopexin | P02790 | NFPSPVDAAFR | 62.88 | 11998.45 | Yes | Yes | 99 |
| Apolipoprotein D | P05090 | NILTSNNIDVK | 39.45 | 2533.48 | Yes | Yes | 99 |
| Insulin-like growth factor-binding protein complex acid labile subunit | P35858 | NLIAAVAPGAFLGLK | 18.59 | 111.16 | Yes | Yes | 99 |
| Aromatase | P11511 | NMLEMIFTPR | 54.21 | 7.61 | Yes | No | 0 |
| Calcitonin gene-related peptide 1 | P06881 (only in canonical sequence) | NNFVPTNVGSK | 4.61 | -0.16 | No | No | 0 |
| Vascular cell adhesion protein 1 | P19320 | NTVISVNPSTK | 45.23 | 28.25 | Yes | Yes | 59 |
| Extracellular matrix protein 1 | Q16610 | NVALVSGDTENAK | 25.74 | 60.15 | Yes | Yes | 99 |
| Ferritin heavy chain | P02794 | NVNQSLLELHK | 2.85 | 1.22 | Yes | No | 8 |
| Tetranectin | P05452 | NWETEITAQPDGGK | 72.16 | 177.62 | Yes | Yes | 99 |
| Alpha-1-acid glycoprotein 1 | P02763 | NWGLSVYADKPETTK | 110.45 | 11133.51 | Yes | Yes | 99 |
| Ig kappa chain V-IV region | P06312/P01625/P06313/P06314 | NYLAWYQQKPGQPPK | 23.34 | 1350.47 | Yes | Yes | 99 |
| Complement C1q subcomponent subunit A | P02745 | PAFSAIR | 14.96 | 209.96 | Yes | Yes | 99 |
| Claudin-5 | O00501 | PDLSFPVK | 18.09 | 5.79 | Yes | No | 0 |
| Nucleoside diphosphate kinase | P15531 | PFFAGLVK | 18.59 | 4.45 | Yes | No | 0 |
| Glutathione peroxidase 3 | P22352 | QEPGENSEILPTLK | 9.39 | 124.28 | Yes | Yes | 99 |
| Coagulation factor IX | P00740 | SALVLQYLR | 50.82 | 59.96 | Yes | Yes | 99 |
| Galectin-3-binding protein | Q08380 | SDLAVPSELALLK | 61.06 | 25.48 | Yes | Yes | 18 |
| Complement component C8 β chain | P07358 | SDLEVAHYK | 13.23 | 169.44 | Yes | Yes | 99 |
| Platelet endothelial cell adhesion molecule | P16284 | SELVTVTESFSTPK | 45.65 | 19.97 | Yes | No | 15 |
| Apolipoprotein F | Q13790 | SGVQQLIQYYQDQK | 15.96 | 744.33 | Yes | Yes | 99 |
| Biotinidase | P43251 | SHLIIAQVAK | 10.63 | 86.48 | Yes | Yes | 99 |
| Carboxypeptidase N catalytic chain | P15169 | SIPQVSPVR | 4.88 | 121.56 | Yes | Yes | 99 |
| Inter-alpha-trypsin inhibitor heavy chain H2 | P19823 | SLAPTAAAK | 29.99 | 1492.53 | Yes | Yes | 99 |
| Lumican | P51884 | SLEDLQLTHNK | 56.45 | 365.12 | Yes | Yes | 99 |
| Fatty acid-binding protein heart | P05413 | SLGVGFATR | 7.91 | -0.51 | No | No | 0 |
| Keratin type I cytoskeletal 10 | P13645 | SLLEGEGSSGGGGR | 89.47 | 15.93 | Yes | No | 0 |
| Occludin | Q16625 | SLQSELDEINK | 44.54 | 9.52 | Yes | Yes | 2 |
| Apolipoprotein A-II | P02652 | SPELQAEAK | 111.99 | 12871.75 | Yes | Yes | 99 |
| Inter-alpha-trypsin inhibitor heavy chain H4 | Q14624 | SPEQQETVLDGNLIIR | 54.46 | 2051.58 | Yes | Yes | 99 |
| Hepatocyte growth factor-like protein | P26927 | SPLNDFQVLR | 50.1 | 18.8 | Yes | No | 11 |
| CD40 ligand | P29965 | SQFEGFVK | 64.36 | 11.31 | Yes | No | 0 |
| Phosphatidylcholine-sterol acyltransferase | P04180 | SSGLVSNAPGVQIR | 12.44 | 207.51 | Yes | Yes | 99 |
| Complement factor H | P08603 | SSQESYAHGTK | 32.66 | 2244.41 | Yes | Yes | 99 |
| Thrombomodulin | P07204 | SSVAADVISLLLNGDGGVGR | 3.82 | 1.38 | Yes | No | 12 |
| Alpha-1-antitrypsin | P01009 | SVLGQLGITK | 50.59 | 31294.28 | Yes | Yes | 99 |
| Attractin | O75882 | SVNNVVVR | 3.68 | 104.75 | Yes | Yes | 99 |
| Actin alpha cardiac muscle 1 | P62736 | SYELPDGQVITIGNER | 74.6 | 69.55 | Yes | Yes | 70 |
| Vascular non-inflammatory molecule 3 | Q9NY84 | TETPVSK | 123.25 | 14.72 | Yes | No | 0 |
| Complement C3 | P01024 | TGLQEVEVK | 64.08 | 5940.55 | Yes | Yes | 99 |
| Mannan-binding lectin serine protease 1 | P48740 | TGVITSPDFPNPYPK | 62.82 | 38.71 | Yes | Yes | 67 |
| Fibulin-1 | P23142 | TGYYFDGISR | 17.9 | 127.3 | Yes | Yes | 99 |
| Complement factor D | P00746 | THHDGAITER | 17.66 | 65.12 | Yes | Yes | 99 |
| Endothelial protein C receptor | Q9UNN8 | TLAFPLTIR | 18.5 | 11.48 | Yes | Yes | 74 |
| Heparin cofactor 2 | P05546 | TLEAQLTPR | 32.72 | 936.78 | Yes | Yes | 99 |
| Glutathione S-transferase P | P09211 | TLGLYGK | 701.19 | 112.92 | Yes | No | 0 |
| Keratin type I cytoskeletal 9 | P35527 | TLLDIDNTR | 31.44 | 17.79 | Yes | Yes | 30 |
| Complement C1s subcomponent | P09871 | TNFDNDIALVR | 54.77 | 408.68 | Yes | Yes | 99 |
| N(G) N(G)-dimethylarginine dimethylaminohydrolase 1 | O94760 | TPEEYPESAK | 73.96 | 13.95 | Yes | No | 0 |
| Coagulation factor XI | P03951 | TSESGLPSTR | 19.9 | 58.25 | Yes | Yes | 99 |
| Sex hormone-binding globulin | P04278 | TSSSFEVR | 20.27 | 51.56 | Yes | Yes | 97 |
| Β-nerve growth factor | P01138 | TTATDIK | 17.91 | 4.76 | Yes | No | 14 |
| P-selectin | P16109 | TWTWVGTK | 14.53 | 5.22 | Yes | No | 23 |
| Stromelysin-1 | P08254 | TYFFVEDK | 66.5 | 10.16 | Yes | No | 0 |
| Apolipoprotein C-II | P02655 | TYLPAVDEK | 9.05 | 1891.55 | Yes | Yes | 99 |
| Apolipoprotein L1 | O14791 | VAQELEEK | 25.07 | 478.75 | Yes | Yes | 99 |
| Myeloperoxidase | P05164 | VFFASWR | 45.74 | 7.48 | Yes | No | 0 |
| Complement C5 | P01031 | VFQFLEK | 7.46 | 258.98 | Yes | Yes | 99 |
| Plasminogen activator inhibitor 1 | P05121 | VFQQVAQASK | 55.43 | 22.26 | Yes | No | 12 |
| Complement factor I | P05156 | VFSLQWGEVK | 127.75 | 462.08 | Yes | Yes | 99 |
| Hemoglobin subunit alpha | P69905 | VGAHAGEYGAEALER | 35.54 | 688.74 | Yes | Yes | 99 |
| Complement C4-B | P0C0L5 | VGDTLNLNLR | 10.24 | 3154.49 | Yes | Yes | 99 |
| Kallistatin | P29622 | VGSALFLSHNLK | 43.81 | 73.2 | Yes | Yes | 99 |
| Carbonic anhydrase 1 | P00915 | VLDALQAIK | 5.53 | 51.8 | Yes | Yes | 99 |
| Vitamin D-binding protein | P02774 | VLEPTLK | 50.38 | 2802.74 | Yes | Yes | 99 |
| Complement C4-A | P0C0L4 | VLSLAQEQVGGSPEK | 162.28 | 2582.68 | Yes | Yes | 99 |
| Β-2-microglobulin | P61769 | VNHVTLSQPK | 10.58 | 168.57 | Yes | Yes | 99 |
| Calponin-1 | P51911 | VNVGVK | 12.39 | 0 | No | No | 0 |
| Fibrinogen alpha chain | P02671 | VQHIQLLQK | 95.22 | 21661.87 | Yes | Yes | 99 |
| Coagulation factor VII | P08709 | VSQYIEWLQK | 22.12 | 6.09 | Yes | Yes | 18 |
| Ig mu heavy chain disease protein | P01871 | VSVFVPPR | 41.05 | 4784.06 | Yes | Yes | 99 |
| Hyaluronan-binding protein 2 | Q14520 | VVLGDQDLK | 12.34 | 184.88 | Yes | Yes | 99 |
| Metalloproteinase inhibitor 4 | Q99727 | VVPASADPADTEK | 7.72 | 1.76 | Yes | No | 0 |
| Tissue-type plasminogen activator | P00750 | VVPGEEEQK | 71.5 | 10.65 | Yes | No | 0 |
| Vascular endothelial growth factor B | P49765 | VVSWIDVYTR | 5.57 | 0.03 | Yes | No | 0 |
| Complement C1r subcomponent-like protein | Q9NZP8 | VVVHPDYR | 3.9 | 72.6 | Yes | Yes | 99 |
| Vitamin K-dependent protein S | P07225 | VYFAGFPR | 15.85 | 394.12 | Yes | Yes | 99 |
| Mannose-binding protein C | P11226 | WLTFSLGK | 47.64 | 35.81 | Yes | Yes | 67 |
| Mannan-binding lectin serine protease 2 | O00187 | WPEPVFGR | 52.19 | 28.66 | Yes | No | 59 |
| Corticosteroid-binding globulin | P08185 | WSAGLTSSQVDLYIPK | 432.28 | 527.56 | Yes | Yes | 99 |
| Ficolin-3 | O75636 | YAVSEAAAHK | 93.97 | 203.37 | Yes | Yes | 99 |
| Fibrinogen gamma chain | P02679 | YEASILTHDSSIR | 73.45 | 19182.42 | Yes | Yes | 99 |
| Keratin-type II cytoskeletal 2 epidermal | P35908 | YEELQVTVGR | 298.56 | 82.93 | Yes | No | 0 |
| CD44 antigen | P16070 | YGFIEGHVVIPR | 40.29 | 17.52 | Yes | Yes | 28 |
| Gamma-enolase | P09104 | YITGDQLGALYQDFVR | 25.05 | 6.86 | Yes | No | 0 |
| Lactotransferrin | P02788 | YLGPQYVAGITNLK | 22.82 | 5.95 | Yes | No | 0 |
| TBC1 domain family member 10A | Q9BXI6 | YLPGYYSEK | 7.38 | 3.08 | Yes | No | 1 |
| Vasorin | Q6EMK4 | YLQGSSVQLR | 18.75 | 18.28 | Yes | Yes | 98 |
| Cholinesterase | P06276 | YLTLNTESTR | 16.27 | 52.31 | Yes | Yes | 99 |
| E-selectin | P16581 | YTHLVAIQNK | 18.59 | 5.46 | Yes | No | 5 |
| Follistatin-related protein 1/Early endosome antigen 1 | Q12841/Q15075 | YVQELQK | 24.74 | 6.07 | Yes | No | 0 |
| Retinol-binding protein 4 | P02753 | YWGVASFLQK | 56.71 | 1044.35 | Yes | Yes | 99 |
| Melanotransferrin | P08582 | YYDYSGAFR | 0 | 0 | No | Yes | 0 |
| Spermine oxidase | Q9NWM0 | YYSTTHGALLSGQR | 9.11 | 1.92 | Yes | No | 0 |

Abbreviations: LLOQ, lower limit of quantification.

**Supplementary Table 2. Top discriminating proteins in a multivariable logistic regression model**

| **Variable** | **β** | **Range** | **Percentage of the 250 bootstrap samples in which the protein was selected** |
| --- | --- | --- | --- |
| Complement component C7 / P10643 | 0.48 | [-0.35, 1.32] | 54.4 |
| β-Ala-His dipeptidase / Q96KN2 | 0.07 | [-0.66, 0.80] | 34.4 |
| Coagulation factor XII / P00748 | -0.05 | [-0.83, 0.73] | 31.6 |
| Intercellular adhesion molecule 1 / P05362 | 0.58 | [-0.45, 1.61] | 32.4 |
| Coagulation factor X / P00742 | -1.53 ** | [-2.54, -0.52] | 53.6 |
| Lumican / P51884 | 0.93 * | [0.11, 1.74] | 36.0 |
| P-selectin / P16109 | 1.00 ** | [0.26, 1.73] | 33.2 |
| Kallistatin / P29622 | -0.58 | [-1.59, 0.44] | 28.8 |
| β-2-microglobulin / P61769 | -0.16 | [-0.94, 0.62] | 31.6 |
| Coagulation factor VII / P08709 | -1.44 * | [-2.60, -0.28] | 53.6 |

**** p < 0.01; * p < 0.05.**

**
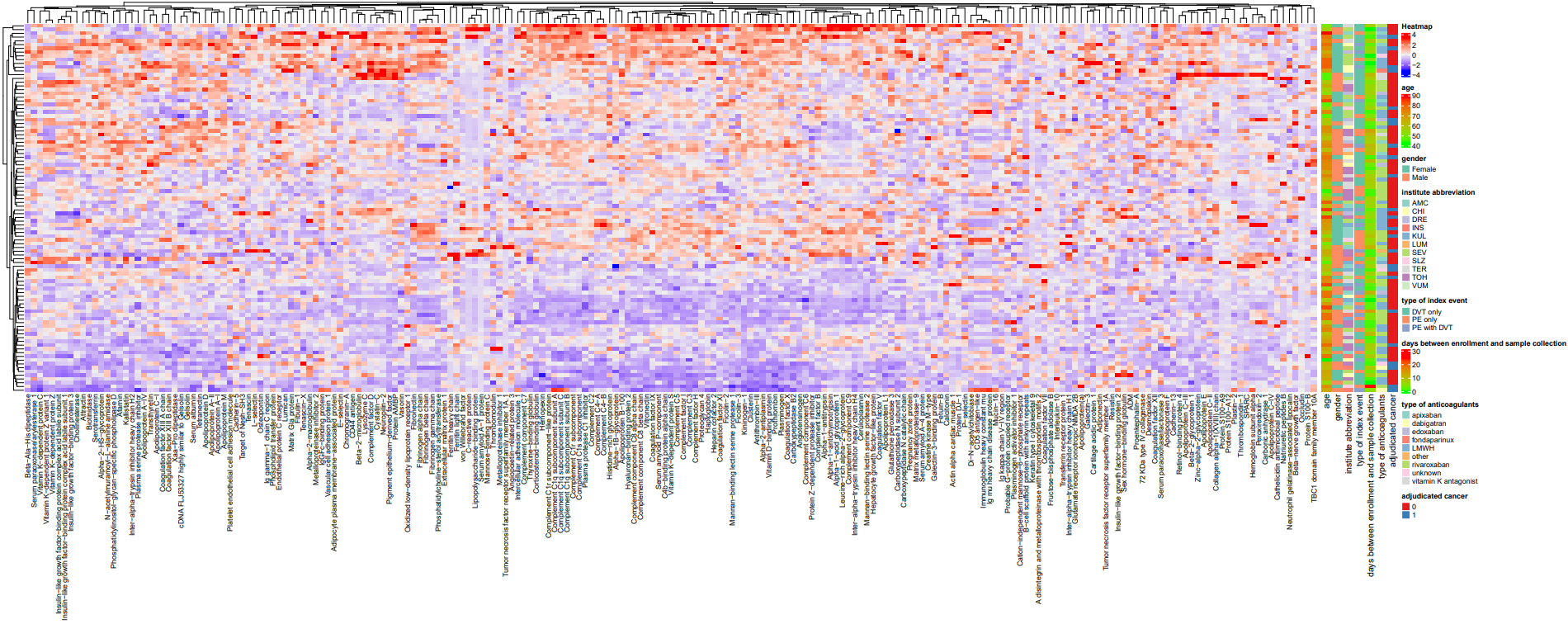
**

**Supplementary Figure 1. Heatmap of the centered and scaled protein abundances in all samples**

Abbreviations: DVT, deep vein thrombosis; PE, pulmonary embolism; LWMH, low-molecular-weight heparin.


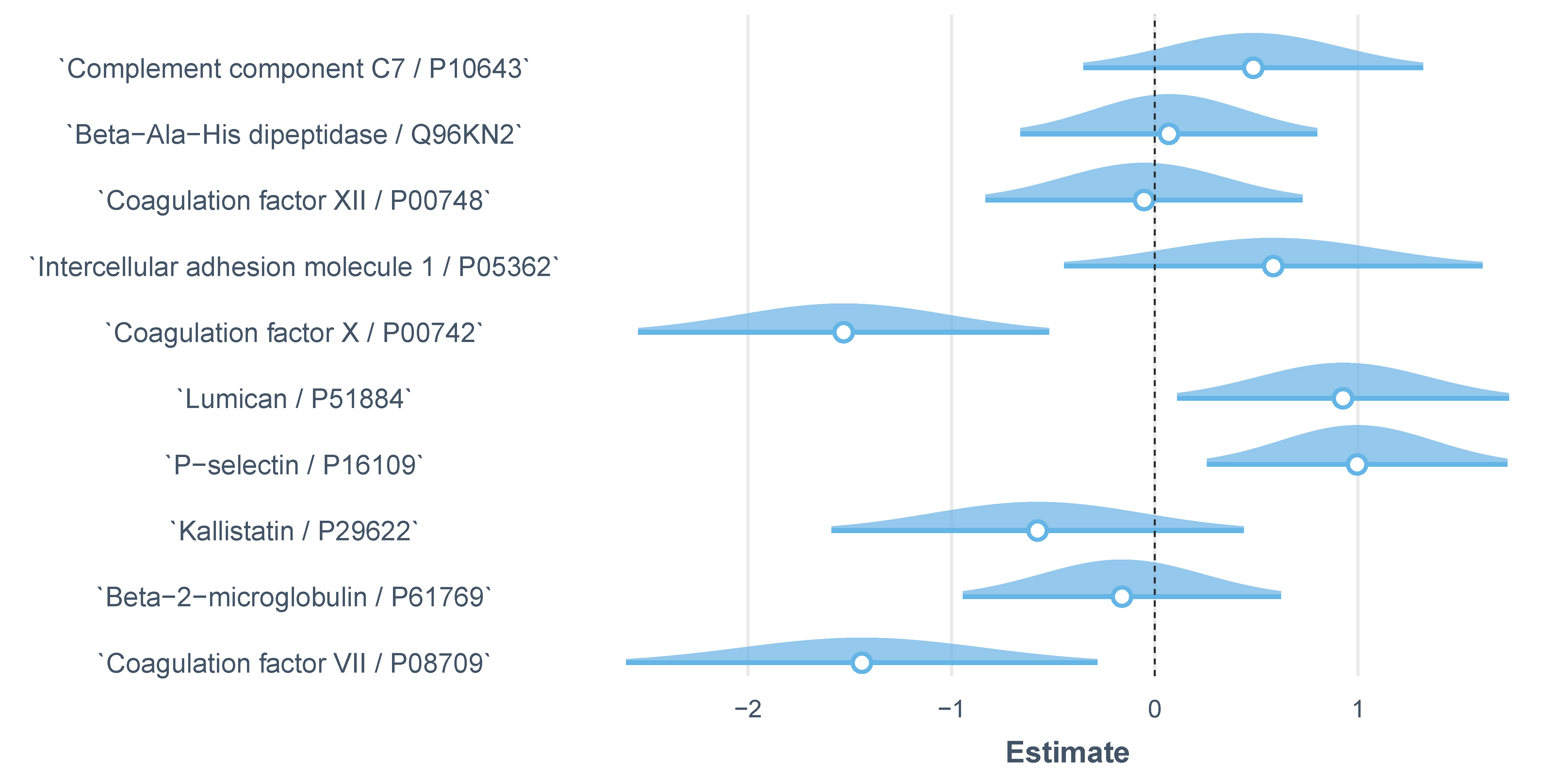
**Supplementary Figure 2. Range of the β coefficient for the top discriminating proteins in a multivariable logistic regression model**

STROBE Statement for “Quantitative proteomics for occult cancer screening in patients with unprovoked venous thromboembolism: results from the prospective PLATO-VTE study”

|  | Item No | Recommendation | Page no. |
| --- | --- | --- | --- |
| **Title and abstract** | 1 | (*a*) Indicate the study’s design with a commonly used term in the title or the abstract | 1,4 |
|  |  | (*b*) Provide in the abstract an informative and balanced summary of what was done and what was found | 4 |
| Introduction | | |  |
| Background/rationale | 2 | Explain the scientific background and rationale for the investigation being reported | 5 |
| Objectives | 3 | State specific objectives, including any prespecified hypotheses | 5 |
| Methods | | |  |
| Study design | 4 | Present key elements of study design early in the paper | 5,6 |
| Setting | 5 | Describe the setting, locations, and relevant dates, including periods of recruitment, exposure, follow-up, and data collection | 5-7 |
| Participants | 6 | (*a*) Give the eligibility criteria, and the sources and methods of selection of participants. Describe methods of follow-up | 5,6 |
|  |  | (*b*) For matched studies, give matching criteria and number of exposed and unexposed | NA |
| Variables | 7 | Clearly define all outcomes, exposures, predictors, potential confounders, and effect modifiers. Give diagnostic criteria, if applicable | 6,7 |
| Data sources/ measurement | 8* | For each variable of interest, give sources of data and details of methods of assessment (measurement). Describe comparability of assessment methods if there is more than one group | 5-7 and Supplemental Methods |
| Bias | 9 | Describe any efforts to address potential sources of bias | 7 |
| Study size | 10 | Explain how the study size was arrived at | 5, 6 |
| Quantitative variables | 11 | Explain how quantitative variables were handled in the analyses. If applicable, describe which groupings were chosen and why | 6,7 |
| Statistical methods | 12 | (*a*) Describe all statistical methods, including those used to control for confounding | 6,7 |
|  |  | (*b*) Describe any methods used to examine subgroups and interactions | 7 |
|  |  | (*c*) Explain how missing data were addressed | 6 |
|  |  | (*d*) If applicable, explain how loss to follow-up was addressed | NA |
|  |  | (*e*) Describe any sensitivity analyses | NA |
| Results | | |  |
| Participants | 13* | (a) Report numbers of individuals at each stage of study—eg numbers potentially eligible, examined for eligibility, confirmed eligible, included in the study, completing follow-up, and analysed | 7 |
|  |  | (b) Give reasons for non-participation at each stage | NA |
|  |  | (c) Consider use of a flow diagram | NA |
| Descriptive data | 14* | (a) Give characteristics of study participants (eg demographic, clinical, social) and information on exposures and potential confounders | 7, Table 1 |
|  |  | (b) Indicate number of participants with missing data for each variable of interest | Table 1 |
|  |  | (c) Summarise follow-up time (eg, average and total amount) | 7 |
| Outcome data | 15* | Report numbers of outcome events or summary measures over time | 7,8 |
| Main results | 16 | (*a*) Give unadjusted estimates and, if applicable, confounder-adjusted estimates and their precision (eg, 95% confidence interval). Make clear which confounders were adjusted for and why they were included | 7,8 |
|  |  | (*b*) Report category boundaries when continuous variables were categorized | NA |
|  |  | (*c*) If relevant, consider translating estimates of relative risk into absolute risk for a meaningful time period | NA |
| Other analyses | 17 | Report other analyses done—eg analyses of subgroups and interactions, and sensitivity analyses | 8 |
| Discussion | | |  |
| Key results | 18 | Summarise key results with reference to study objectives | 8,9 |
| Limitations | 19 | Discuss limitations of the study, taking into account sources of potential bias or imprecision. Discuss both direction and magnitude of any potential bias | 10 |
| Interpretation | 20 | Give a cautious overall interpretation of results considering objectives, limitations, multiplicity of analyses, results from similar studies, and other relevant evidence | 9,10 |
| Generalisability | 21 | Discuss the generalisability (external validity) of the study results | 10 |
| Other information | | |  |
| Funding | 22 | Give the source of funding and the role of the funders for the present study and, if applicable, for the original study on which the present article is based | 1 |

*Give information separately for exposed and unexposed groups.

Abbreviations: NA, not applicable.
